# The Selection of a Hepatocyte Cell Line Susceptible to *Plasmodium falciparum* Sporozoite Invasion That Is Associated With Expression of Glypican-3

**DOI:** 10.3389/fmicb.2019.00127

**Published:** 2019-02-28

**Authors:** Rebecca E. Tweedell, Dingyin Tao, Timothy Hamerly, Tanisha M. Robinson, Simon Larsen, Alexander G. B. Grønning, Alessandra M. Norris, Jonas G. King, Henry Chun Hin Law, Jan Baumbach, Elke S. Bergmann-Leitner, Rhoel R. Dinglasan

**Affiliations:** ^1^Department of Infectious Diseases and Immunology, Emerging Pathogens Institute, University of Florida, Gainesville, FL, United States; ^2^W. Harry Feinstone Department of Molecular Microbiology and Immunology, Johns Hopkins Malaria Research Institute, Johns Hopkins Bloomberg School of Public Health, Baltimore, MD, United States; ^3^Malaria Vaccine Branch, Walter Reed Army Institute of Research, Silver Spring, MD, United States; ^4^Computational BioMedicine Lab, Department of Mathematics and Computer Science, University of Southern Denmark, Odense, Denmark; ^5^Department of Biochemistry, Molecular Biology, Entomology & Plant Pathology, Mississippi State University, Starkville, MS, United States; ^6^Chair of Experimental Bioinformatics, TUM School of Life Sciences Weihenstephan, Technical University of Munich, Munich, Germany

**Keywords:** malaria, *Plasmodium falciparum*, liver stage, *in vitro* model, omics, glypican-3, hepatocyte

## Abstract

*In vitro* studies of liver stage (LS) development of the human malaria parasite *Plasmodium falciparum* are technically challenging; therefore, fundamental questions about hepatocyte receptors for invasion that can be targeted to prevent infection remain unanswered. To identify novel receptors and to further understand human hepatocyte susceptibility to *P. falciparum* sporozoite invasion, we created an optimized *in vitro* system by mimicking *in vivo* liver conditions and using the subcloned HC-04.J7 cell line that supports mean infection rates of 3–5% and early development of *P. falciparum* exoerythrocytic forms—a 3- to 5-fold improvement on current *in vitro* hepatocarcinoma models for *P. falciparum* invasion. We juxtaposed this invasion-susceptible cell line with an invasion-resistant cell line (HepG2) and performed comparative proteomics and RNA-seq analyses to identify host cell surface molecules and pathways important for sporozoite invasion of host cells. We identified and investigated a hepatocyte cell surface heparan sulfate proteoglycan, glypican-3, as a putative mediator of sporozoite invasion. We also noted the involvement of pathways that implicate the importance of the metabolic state of the hepatocyte in supporting LS development. Our study highlights important features of hepatocyte biology, and specifically the potential role of glypican-3, in mediating *P. falciparum* sporozoite invasion. Additionally, it establishes a simple *in vitro* system to study the LS with improved invasion efficiency. This work paves the way for the greater malaria and liver biology communities to explore fundamental questions of hepatocyte-pathogen interactions and extend the system to other human malaria parasite species, like *P. vivax*.

## Introduction

Malaria is a devastating disease that affects over 200 million people each year and causes approximately 445,000 deaths, mainly among young children (WHO, 2017). *Plasmodium falciparum* is one of the major parasites responsible for morbidity and mortality. This parasite is transmitted to humans as a sporozoite through the bite of an infected female anopheline mosquito during blood feeding. From the bite site, the sporozoite makes its way to the liver, where it infects a hepatocyte (Yamauchi et al., 2007). The infection of hepatocytes causes no clinical symptoms, allowing the parasite to develop and multiply to prepare for the invasion of red blood cells, which results in clinical disease (Phillips and Pasvol, 1992; Vaughan et al., 2008). The LS is a crucial step in the parasite’s life cycle, as it establishes vertebrate infection; however, studying *P. falciparum* LS development has been technically challenging. Studies carried out using primary human hepatocytes face the obstacles of these cells not propagating in culture, being in short supply, and producing highly variable infection rates (0.13–2%) (Smith et al., 1984; Mazier et al., 1985; Vaughan et al., 2008; Roth et al., 2018). While recent work has improved the utility of primary cells, this system requires the screening of different lots of primary cells to identify those that support sporozoite invasion and development, limiting widespread use (Roth et al., 2018). Development of a suitable alternative to using primary human hepatocytes for the study of the *P. falciparum* LS is desirable.

*P. falciparum* and *P. vivax* sporozoites can infect and develop in the human hepatocarcinoma cell line HC-04, but infection efficiency remains marginal, customarily between 0.13% and 0.7–1% for *P. falciparum* (Sattabongkot et al., 2006; Mikolajczak et al., 2011; Tao et al., 2014). HC-04 is a spontaneously immortalized cell line that was isolated from normal human hepatocytes (Prachumsri and Yimamnuaychok, 2002). Recent analyses of this line suggest that, unlike other commonly used hepatocarcinoma cell lines, like HepG2, HC-04 exhibits more plasticity and a greater propensity to recover its epithelial characteristics (Tao et al., 2014), opening the possibility to create a sporozoite invasion system based on this line. Such a system would greatly improve the ability to perform high-throughput drug screening for LS compounds (malERA Refresh Consultative Panel on Basic Science and Enabling Technology, 2017) and study the biology of the LS in a homogeneous population of cells that can be distributed as a shared resource to laboratories all over the world.

Technical limitations of studying the mammalian *Plasmodium* LS have hampered the identification of proteins involved in sporozoite host cell invasion and infection and left the process poorly understood for *P. falciparum*. Most studies have focused on the rodent *Plasmodium* species. However, differences in sporozoite host cell tropism and the lack of conservation of hepatocyte surface receptors necessary for invasion suggest significant differences exist between these species and *P. falciparum* (Kaushansky and Kappe, 2015); focusing studies on rodent parasites alone can cause essential factors for *P. falciparum* sporozoite invasion to be missed or overlooked. Using various model systems, it has been demonstrated that SCARB1 (Rodrigues et al., 2008), SDC2 (Frevert et al., 1993), EphA2 (Kaushansky et al., 2015), LRP1 (Shakibaei and Frevert, 1996), CD81 (Silvie et al., 2003), and c-Met (*P. berghei* only; Kaushansky and Kappe, 2011) can each play a role as hepatocyte receptors for sporozoite invasion and infection, but the molecular invasion mechanism for *P. falciparum* remains largely unknown. Additionally, the steps of LS development following sporozoite invasion are not well defined for *P. falciparum.* These knowledge gaps in LS biology, along with the difficulty of implementing high-throughput screens for this stage, have been major roadblocks in identifying much needed drug targets and vaccine candidates (Derbyshire et al., 2012; Longley et al., 2015).

Herein, we applied comparative proteomics and RNA-seq approaches to identify surface molecules and pathways from *P. falciparum* sporozoite invasion-susceptible and invasion-resistant hepatocarcinoma cell lines that are potentially important for *P. falciparum* sporozoite invasion. We further investigated GPC3 as a putative receptor mediating sporozoite invasion of hepatocytes using a robust platform for *P. falciparum* sporozoite invasion of and early exoerythrocytic form development in a hepatocarcinoma line. This platform effectively overcomes the cost barrier and high variability of primary human tissue and expands the utility of *in vitro* culture for LS studies. The comparative multi-omics dataset identifies other important host cell pathways that may also influence hepatocyte susceptibility to *P. falciparum* sporozoite invasion, spurring hypothesis generation and testing by the greater scientific community.

## Materials and Methods

### Ethics Statement

The human blood used for the mosquito blood meal was collected from a pool of pre-screened donors under an IRB-approved protocol at Johns Hopkins University (Protocol NA00019050) or obtained commercially from anonymous donors through Interstate Blood Bank, making informed consent not applicable. The original isolation of hepatocytes to establish the HC-04 cell line was approved by the Ethics Committee of the Thai Ministry of Public Health and the Human Subjects Research Review Board of the United States Army.

### Cell Line Maintenance

HC-04 (kindly provided by the Naval Medical Research Center through Dr. Eileen Villasante and originally isolated from the healthy fringe of a hepatoma patient undergoing therapeutic surgery at Ramathibodi Hospital [Bangkok, Thailand]), HC-04 subcloned cell lines, and HepG2 (kindly provided by Dr. Photini Sinnis) were maintained in T75 flasks in IMDM (Life Technologies, Carlsbad, CA, United States) supplemented with 5% HIFBS, 200 units/mL penicillin, and 200 μg/mL streptomycin (Corning, Corning, NY, United States). From here on, we refer to both parental and subcloned cells as HC-04. Cells were split at a 90% confluent culture by digesting the monolayer in 5 mL of 0.05% trypsin-EDTA for 10 min or until cells lifted. The cell suspension was collected in a conical tube and centrifuged at 700 × *g* for 7 min to ensure pelleting of the cells. Trypsin was removed, and cells were resuspended in media for plating, then plated at a 1:10 dilution in a new T75 flask in fresh IMDM with 5% HIFBS, 200 units/mL penicillin, and 200 μg/mL streptomycin for maintenance of the line; cells were plated as needed for other uses (as outlined below). The parental HC-04 line was cleared of mycoplasma contamination. HepG2 and all HC-04-derived cell lines also were monitored for mycoplasma contamination by microscopy and treated with BM-Cyclin (Roche, Basel, Switzerland) if needed to clear any contamination before frozen stocks were created.

### Sporozoite Generation and Isolation

*Anopheles stephensi* (days 6–10) mosquitoes were fed a blood meal containing *P. falciparum* NF54 (WRAIR) gametocytes (diluted to 0.3% stage V gametocytemia) on day 1 of each experiment. On day 18 post-mosquito feed, 3 mosquitoes per well of cells to be infected were dissected to obtain salivary glands; the salivary glands were kept in M199 medium with 1% w/v heat inactivated BSA in a 1.5 mL tube on ice during the dissection (Vanderberg, 1974; Kebaier and Vanderberg, 2010; Lupton et al., 2015). The tube of salivary glands was spun down at 1200 × *g* for 3 min at room temperature. The salivary gland pellet was gently crushed with a plastic pestle in the 1.5 mL tube and vortexed 3 × 3 s to suspend the salivary gland contents in the M199 medium. Using a 26-gauge needle heated by a Bunsen burner flame, a hole was poked in the bottom of a 500 μL tube. Approximately 300 μL of glass wool (Supelco, Sigma-Aldrich) was added to the 500 μL tube, ensuring the glass wool fit easily at the bottom of the tube. The 500 μL tube containing glass wool was placed in a 1.5 mL collecting tube. The crushed salivary gland mixture was filtered through this 500 μL tube with glass wool approximately 200 μL at a time, spinning at 1200 × *g* for 3 s for each 200 μL fraction at room temperature. After each spin, the liquid accumulated in the 1.5 mL collecting tube was transferred to a fresh 1.5 mL tube on ice; all fractions were combined into one 1.5 mL tube on ice. The glass wool was washed with 200 μL PBS, spinning at 1200 × *g* for 10 s at room temperature; the liquid accumulated in the 1.5 mL collecting tube was transferred to the 1.5 mL tube on ice that contained the other fractions. Sporozoites were counted using a hemocytometer.

### Plating HC-04 for Infection

On day 17 post-mosquito feed, 12 mm diameter coverslips were coated in the wells of a 24-well plate with 0.01% w/v collagen in PBS and incubated under UV light at room temperature for 1 h. The collagen was removed, and coverslips were washed once with PBS. HC-04 (50,000 per well) were plated in 24-well plates on the collagen-coated coverslips in 500 μL media. Media used were “culture media” (CM): equal volumes MEM and F12 supplemented with 10% HIFBS, 15 mM HEPES, 20 mM sodium bicarbonate, 15 μM phenol red, 200 units/mL penicillin, and 200 μg/mL streptomycin (Sattabongkot et al., 2006; Cui et al., 2009); and “DMEM-NoGlc:” DMEM without Glc (Life Technologies), supplemented with 1 mM sodium pyruvate (Life Technologies), 1% FBS (Corning), 200 units/mL penicillin, and 200 μg/mL streptomycin. Additional supplementations of the DMEM-NoGlc that were tested were 1 × MEM amino acids without L-glutamine (Sigma-Aldrich) and chemically defined lipid mixture 1 (Sigma-Aldrich, St. Louis, MO, United States; containing 4 ng/mL arachidonic acid; 20 ng/mL linoleic, linolenic, myristic, oleic, palmitic, and stearic acids; 0.44 μg/mL cholesterol, 4.4 μg/mL Tween-80, 140 ng/mL tocopherol acetate, and 200 μg/mL pluronic F-68).

### Infection of HC-04 With Sporozoites

For the ILSDA with the 2A10 antibody, which recognizes the NANP repeat on the *P. falciparum* CSP (Nardin et al., 1982; Burkot et al., 1991), 50,000 sporozoites per well of HC-04 to be infected were co-incubated with antibody (anti-CSP antibody 2A10, MRA183A, Malaria Research & Reference Reagent Resource Center [MR4], Bei Resources); or mouse control mAb clone 1D9, diluted in 100 μL of media and incubated for 20 min at room temperature prior to their addition to the HC-04 in 0.6 mL of DMEM-NoGlc. Unbound antibody was not removed before addition of sporozoites to the hepatocyte culture. For the ILSDA with the anti-GPC3 antibody, HC-04 were treated with anti-GPC3 antibody (Fisher Scientific, Hampton, NH, United States; MAB2119; 10 μg/mL) for 15 min at 37°C prior to the addition of sporozoites. For studies of liver-stage biology, 50,000 sporozoites were directly added to each well containing HC-04 in 500 μL fresh DMEM-NoGlc. For Glc supplementation upon sporozoite addition, 15 mM D-Glc was added to the 500 μL DMEM-NoGlc media containing the sporozoites prior to addition to cells. In all cases, after sporozoite addition to the HC-04 cells, the plate was gently swirled five times by hand, and then spun at 50 × *g* for 2 min at room temperature. The plate was then incubated at 37°C in an incubator (5% CO_2_) for 10 min. This swirling and spinning was repeated two more times. Following the third centrifugation, the plate was incubated at 37°C for 24 h under standard ‘normoxia’ (5% CO_2_) or under hypoxia in a hypoxia chamber (Billups-Rothenberg, San Diego, CA, United States) containing 5% oxygen to recapitulate the partial pressure of oxygen in the liver (30–75 mmHg; Wolfle et al., 1983), which is significantly lower than that usually encountered by cells in culture (110–130 mmHg) (Ng et al., 2014). Infections and ILSDAs were performed with at least three biological replicates and repeated several times with different pools of sporozoites to ensure reproducibility. The infection protocol was carried out by two independent laboratories to confirm invasion rates. In the context of these assays, invasion is defined as successful sporozoite entry into a hepatocyte within 24 h in culture.

### Fixing and Staining

After incubating sporozoites with the HC-04 cells for 24 h, media was removed, and the coverslips were washed with 500 μL PBS. Coverslips were then transferred to a new 24-well plate containing 500 μL PBS. The cells were fixed in 110 μL 4% paraformaldehyde for 10 min at room temperature. Paraformaldehyde was removed, and coverslips were washed with 500 μL PBS, then blocked in 500 μL 5% HIFBS in PBS for 30 min at room temperature on a shaker. HIFBS solution was removed, and 110 μL primary antibody (0.89 μg/mL 2A10 monoclonal antibody in PBS) was added. Primary antibody was incubated with the cells for 20 min at room temperature on a shaker, then removed, and coverslips were washed with 500 μL PBS, 4 × 5 min at room temperature on a shaker. Next, 110 μL secondary antibody (1 μg/mL Alexa Fluor 488 anti-mouse [Life Technologies, A-11001] in PBS) was added and incubated for 20 min in the dark at room temperature on a shaker. The secondary antibody was removed, and coverslips were washed with 500 μL PBS, 2 × 5 min at room temperature on a shaker. Coverslips were then washed with 500 μL PBS + 0.1% Tween 20, 2 × 5 min at room temperature on a shaker. Then 110 μL primary antibody (0.89 μg/mL 2A10 in PBS + 0.1% Tween 20) was added and incubated for 20 min at room temperature on a shaker. Primary antibody was removed, and coverslips were washed with 500 μL PBS + 0.1% Tween 20, 4 × 5 min at room temperature on a shaker. Then 110 μL secondary antibody (1 μg/mL Alexa Fluor 594 [Life Technologies, A-11005] anti-mouse in PBS + 0.1% Tween 20) was added and incubated for 20 min at room temperature on a shaker. Secondary antibody was removed, and coverslips were washed with 500 μL PBS + 0.1% Tween 20, 4 × 5 min at room temperature on a shaker. Finally, 110 μL DAPI (5 μg/mL in PBS + 0.1% Tween 20) was added and incubated for 10 min at room temperature on a shaker. DAPI was removed, and coverslips were washed with 500 μL PBS + 0.1% Tween 20, 4 × 5 min at room temperature on a shaker. Coverslips were then mounted to slides on a drop of Aqua Poly/Mount (Polysciences, Inc., Warrington, PA, United States). The coverslips were allowed to set for at least 12 h in the dark at 4°C before visualization.

Immunofluorescence assays of hepatocyte proteins were performed similarly. After culturing for 24 h in the appropriate media, cells were washed with PBS and fixed in 4% paraformaldehyde for 10 min at room temperature. After fixing, cells were washed with 500 μL PBS, then blocked using 5% HIFBS in PBS for 30 min at room temperature on a shaker. HIFBS solution was removed, and 110 μL primary antibody was added overnight at 4°C; primary antibody was made using a 1:500 dilution of anti-EphA2 (BioLegend, San Diego, CA, United States; clone SHM16) or anti-GPC3 (Fisher Scientific, MAB2119) in PBS. Cells were then washed with PBS. Secondary antibody (1 μg/mL anti-mouse Alexa Fluor 594 in PBS) was added to the cells for 1 h at room temperature. Cells were then washed with PBS. Finally, DAPI (5 μg/mL in PBS) was added to the cells and incubated for 7 min at room temperature. DAPI was removed, and coverslips were washed with PBS. Coverslips were then mounted to slides on a drop of Aqua Poly/Mount. The coverslips were allowed to set for at least 12 h in the dark at 4°C before visualization.

### Invasion Quantification

Following sporozoite invasion, cells were visualized under a Nikon Eclipse E800 microscope at 40× for invasion quantification or slides were analyzed at 400× magnification using an Olympus BX51 fluorescence microscope and the cellSens software package (Olympus America Inc., Center Valley, PA, United States). Beginning on the left side of the coverslip and moving in a straight line to the right, the number of red sporozoites that are NOT green (these are the sporozoites inside cells) and the number of HC-04 cells in all fields were counted. The invasion rate was calculated using the equation:

Number of sporozoites inside a cellTotal number of HC−04 cells examined×100

### Imaging

For imaging sporozoite invasion and confocal imaging of developing exoerythrocytic forms, cells were visualized using a Nikon 90i microscope at 100× magnification, and images were acquired with a Hamamatsu Orca-ER camera using the Volocity 3D Image Analysis Software with image stacks deconvolved prior to combining and focused along the plane of the parasites. Epifluorescence imaging of exoerythrocytic forms was performed using an Olympus BX-53 upright microscope at 100× magnification. Imaging of hepatocytes following protein staining was performed using either a Zeiss Axioskop 2 microscope with a ProgRes MFcool camera using the ProgRes CapturePro software version 2.10.0.1 or using a BZ-X710 (Keyence, Osaka, Japan) All-in-One Fluorescence Microscope. BZ-X700 Analyzer Software (version 1.31.1) was used for *Z*-stack analyses to yield a single compressed fully focused image at 600× magnification from 9 to 11 planes, with each plane at 0.2 μm thickness.

### Exoerythrocytic Form Development

After initial sporozoite invasion was allowed in the DMEM-NoGlc media for 24 h, the media was removed and replaced with MEM + F12 (1:1 ratio) supplemented with 10% FBS, 200 units/mL penicillin, and 200 μg/mL streptomycin (referred to as MEM + F12) and replaced daily from day 3 post-infection onwards. Cells were also transferred to mild anoxic conditions in “malaria gas” (5% CO_2_, 5% O_2_). Day 5 was selected as the target harvest date as this would allow evaluation of developmental phenotypes without potential loss of signal through rupturing hepatic schizonts, and a system for direct infection of red blood cells within an *in vitro* culture has not yet been developed. Cells were fixed as described above. Anti-merozoite surface protein-1 (MSP-1) primary antibody (mAb 5.2, ATCC, Manassas, VA; AlexaFluor488-conjugated or AlexaFluor594-conjugated) and/or anti-PfHSP70 (StressMarq Biosciences, Victoria, BC, Canada) were used to identify developing exoerythrocytic forms over a 120-h (5-day) period. HSP70 and MSP1 were used either in tandem or independently as they can capture earlier and later developmental transitions, respectively.

### LC-MS/MS Sample Preparation

For LC-MS/MS, cells were grown in T75 flasks in the appropriate media for 24 h, with three biological replicates per growth condition. Cells were then washed three times with cold PBS and treated with 0.01% trypsin at 37°C for 5 min, then scraped from the flask, and pelleted by centrifugation at 800 × *g* for 5 min. The cell pellet was washed twice with cold PBS and pelleted as above. Protein sample preparation for whole proteome analysis of HC-04 grown in CM and DMEM-NoGlc was performed as previously described (Tweedell et al., 2015); sample preparation for membrane-enriched proteome analysis of HC-04 and HepG2 grown in IMDM was performed similarly, with soluble proteins being discarded. Briefly, cell pellets were suspended in 5 mM phosphate buffer (pH 7.4) containing 0.5 mM PMSF (Sigma-Aldrich), 1 mM EDTA, and 1 mM protease inhibitor cocktail (Sigma-Aldrich). Cells were lysed using four cycles of freeze/thaw in liquid nitrogen for 1 min followed by incubation at 37°C for 4 min. The sample was then pelleted by centrifugation at 20,000 × *g* for 5 min at +4°C, and the supernatant containing soluble proteins was transferred to a new tube (whole proteome analysis) or discarded (membrane-enriched proteome analysis). The pellet was washed with ice cold PBS twice and with centrifugation as above. Membrane proteins were solubilized in SDST-lysis buffer (100 mM Tris-HCl, 4% SDS, 100 mM DTT, pH 7.6) and boiled at 95°C for 5 min. The sample was pelleted by centrifugation at 20,000 × *g* for 5 min (+4°C), and the supernatant containing membrane proteins was transferred to a new tube. Soluble and membrane protein fractions were digested using a FASP protocol (Wisniewski et al., 2009) using a 10 kDa molecular weight cutoff filter (EMD Millipore, Burlington, MA, United States). Acidic tryptic peptides were desalted using an offline HPLC C18 column and fraction collector on an Agilent 1260 HPLC system (Agilent Technologies, Santa Clara, CA, United States), then dried by vacuum centrifugation and stored at −20°C until analysis.

### Online 2D LC-MS/MS

The FASP-desalted peptides were dissolved in loading buffer (97.9% water, 2% ACN, and 0.1% FA) and ≈ 20 μg of peptides were injected to our constructed online 2D HPLC-MS/MS system as described previously (Tao et al., 2014). Briefly, one SCX column was integrated into an Agilent LC-MS system comprised of a 1200 LC system coupled to a 6520 QTOF via an HPLC Chip Cube interface. Peptides were loaded into the SCX column for online SCX fractionation in the first dimension. The peptides were then eluted using the autosampler by injecting increasing concentrations of sodium chloride (NaCl) (0, 15, 30, 45, 60, 120, 160, and 300 mM NaCl in 2% ACN/0.1% FA; followed by one injection of 500 mM NaCl in 2% ACN/0.1% FA to wash the column). The salt elution was captured by a C18 enrichment column integrated into the Agilent Polaris-HR-Chip-3C18 chip. For separation in the second dimension, with the valve switched and the HPLC gradient started, the peptides were eluted from the enrichment column and separated by a C18 analytical column. Peptides were eluted from the analytical column using a gradient starting at 97% A (A: 99.9% water, 0.1% FA) at 300 nL/min. The mobile phase was 3–10% B (B: 90% ACN, 9.9% water, 0.1% FA) for 4 min, 10–35% B for 56 min, 35–99% B for 2 min, and maintained at 99% B for 6 min, followed by re-equilibration of the column with 3% B for 10 min. Data-dependent MS acquisition was performed by an Agilent 6520 QTOF. Precursor MS spectra were acquired from *m/z* 315 to 1700, and the top four peaks were selected for MS/MS analysis. Product scans were acquired from *m/z* 50 to 1700 at a scan rate of 1.5 spectra per second. A medium isolation width (≈4 amu) was used, and a collision energy of slope 3.6 V/100 Da with a 2.9 V offset was applied for fragmentation. A dynamic exclusion list was applied with precursors excluded for 0.50 min after two MS/MS spectrum were acquired.

### Database Searching and Label-Free Quantification Analysis

All the LC-MS/MS raw data were converted to Mascot generic Format (.mgf) by Agilent MassHunter Qualitative Analysis B.04.00. Mascot version 2.4.1 was used to search the SwissProt human 2012 protein FASTA sequence database (20,234 sequences) for peptide sequence assignments using the following parameters: precursor ion mass tolerance of 50 ppm and a fragment ion mass tolerance of 0.2 daltons. Peptides were searched using fully tryptic cleavage constraints and up to two internal cleavage sites were allowed for tryptic digestion. Fixed modifications consisted of carbamidomethylation of cysteine. Variable modifications considered were oxidation of methionine residues. The Mascot search results were exported as .DAT format and then imported into the Scaffold software (Version 4.0.4, Proteome Software) for curation, label-free quantification analysis, and visualization. Scaffold’s normalized spectral counting was employed to compare relative protein abundance between HC-04 cell samples grown in CM and HC-04 grown in DMEM-NoGlc in each experiment as the basis for normalization of the spectral counts for all other LC-MS/MS data in that experiment. Overall, protein false discovery rates of less than 1% and peptide false discovery rates of less than 1% were obtained with Scaffold filters, and each protein has ≥2 unique peptides. Proteomics data have been uploaded to the PRIDE database with the dataset identifier PXD008613.

### RNA-Seq Experiments

For RNA-Seq analyses, HC-04 or HepG2 were grown in T75 flasks in the appropriate media for 24 h, with three biological replicates for each cell type and culture condition. Cells were then washed twice in ice cold PBS. Cells were suspended in TRIzol. RNA was prepared following the manufacturer’s protocol. RNA-seq was performed on an Illumina HiSeq3000 (Illumina, San Diego, CA, United States) with 2 × 100 cycles based on the manufacturer’s guidelines.

Total RNA with an OD 260/280 ratio ranging from 1.2 to 2.2 was used to determine the RNA concentration on a Qubit 2.0 Fluorometer (Thermo Fisher, Waltham, MA, United States). RNA quality was assessed using the Agilent 2100 Bioanalyzer. Total RNA with 28S/18S > 1 and RNA integrity number ≥ 7 was used for RNA-seq library construction.

Approximately 500 ng of protein-free and intact total RNA was used for library construction using the reagents provided in the NEBNext Ultra II RNA Library Prep Kit following the manufacturer’s protocol. First, 2 μL of diluted RNA was spiked with ERCC from the kit. Next, mRNA isolation was performed using the NEBNext Poly(A) mRNA Magnetic Isolation module (New England Biolabs, Ipswich, MA, United States). Then RNA was fragmented in a solution containing divalent cations, with incubation at 94°C. Next, first strand cDNA synthesis using reverse transcriptase and random primers was done. Synthesis of double stranded cDNA was done using the second strand master mix provided in the kit, followed by end-repair and dA-tailing. At this point, Illumina adaptors were ligated to the sample. Finally, the library was enriched by 11 cycles of amplification and purified by Agencourt AMPure beads (Beckman Coulter, Pasadena, CA, United States). Barcoded libraries were sized on the bioanalyzer and quantitated by QUBIT. Quantitative PCR was used to validate the library’s functionality, using the KAPA Library Quantification kit (Kapa Biosystem, Wilmington, MA, United States) and monitoring with the Bio-Rad Touch Real-Time PCR Detection System. Individual libraries were pooled equimolarly for sequencing runs.

Sequencing was performed on the Illumina HiSeq3000 instrument using the clustering and sequencing reagents provided by Illumina. Paired-end, 2 × 100 cycles runs require the combination of reagents from the 150 cycles and the 50 cycles kits. Sequencing reactions were set up using 5 μL of library (2.5 nM). Libraries were first denatured with 5 μL 0.1 N NaOH for 8 min at room temperature. This was followed by neutralization with 5 μL of 200 mM Tris (pH 7.5) and mixing with 35 μL of the ExAmp reagents (contained in the PE-410-1001 clustering kit) according to the manufacturer’s protocol. Samples were clustered in the cBot clustering station using the “HiSeq 3000/4000 HD Exclusion Amp v1.0” protocol. Runs were set by choosing the ‘Generate FASTQ only’ workflow in the HiSeq Control Software v3.3.76 in the computer station that runs the HiSeq3000 sequencing machine (Illumina). Under these run conditions, the cluster pass-filter was 70–75%, with a yield of 300–325 million pass-filter reads per lane. The % ≥ Q30 score was typically above 95%. The reads that passed Illumina quality control filtering were used as raw data for further bioinformatics analysis.

The RNA library construction and HiSeq 3000 sequencing run were performed at the Interdisciplinary Center for Biotechnology Research Gene Expression & Genotyping Core, University of Florida. Reads were trimmed using Trimmomatic 0.36 (Bolger et al., 2014). All leading and trailing bases with quality below 3 were trimmed. Reads were scanned from the 5′ end toward the 3′ end using a sliding window of size 4 and were cut when the average quality within the window dropped below 15. Reads shorter than 40 bases after trimming were discarded. Adapters were removed from reads using the TruSeq3 adapter library provided with Trimmomatic.

Reads were aligned against the GRCh38 reference genome with gene annotations from GENCODE release 26 (both obtained April 6, 2017) using STAR 2.5.3a (Dobin et al., 2013). Gene expression estimates were computed with the “–quantMode GeneCounts” flag, giving the unambiguous, unique number of reads for each gene. The GeneCounts mode is equivalent to running htseq-count with the union overlap resolution mode and discarding ambiguous reads.

Differentially expressed genes were identified using DESeq2 1.28.0 (Love et al., 2014). We compared all possible pairs of the three different cell lines within the same media with default parameters provided by DESeq2. A linear model was fit to each gene with cell line as the dependent variable and all gene expression estimates as independent variables. For each gene set, we performed a statistical enrichment test to test whether the fold changes within the gene set were significantly different from the distribution of fold changes over all genes. A *P*-value was computed using a two-sided Mann–Whitney *U* test. RNA-seq data have been uploaded to the ArrayExpress database with the accession number E-MTAB-6919.

### *In silico* Analyses

For analysis of pathways and protein relationships, protein UniProt ID’s were uploaded to DAVID Bioinformatics Resource 6.7 (National Institute of Allergy and Infectious Disease, NIH). Human GPC3 was submitted to STRING v10.5^1^ for ad hoc protein interaction network analyses (Szklarczyk et al., 2017). *K*-means cluster analysis was used to cluster the nodes into three clusters. The number of nodes was expanded by one level to a total of 21 nodes, in order to show a more complete interaction network, and a summary view of the network was exported.

## Results

### Controlling *in vitro* Culture Conditions to Mimic the *in vivo* Liver Microenvironment and Sub-Cloning the HC-04 Cell Line Establishes an Optimized Model for *P. falciparum* Sporozoite Invasion of Hepatocytes

**FIGURE 1:**
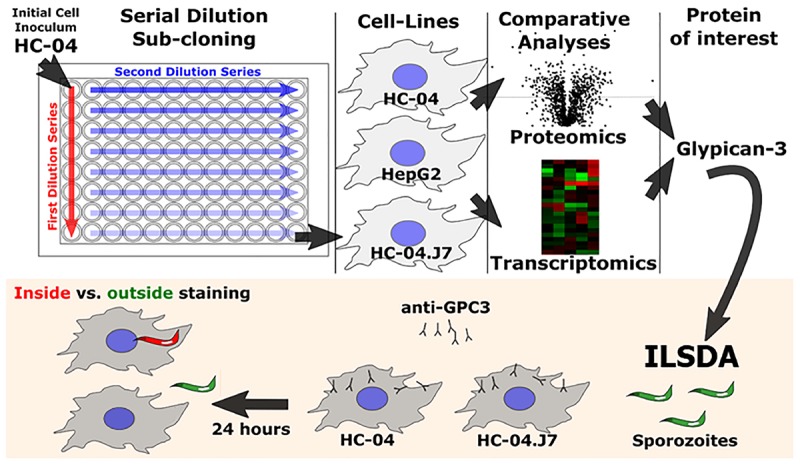
Schematic of the overall experimental approach utilized in this study. ILSDA, inhibition of liver stage development assay.

Identifying hepatocyte receptors and pathways for *P. falciparum* sporozoite invasion first required the establishment of an *in vitro* platform that could be used as the basis for comparing invasion susceptible and non-susceptible cell lines (Figure 1).

**FIGURE 2:**
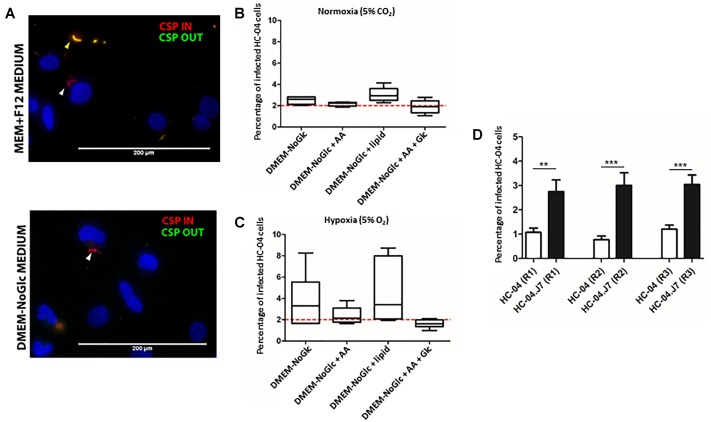
Establishment of an optimized *in vitro* system for *Plasmodium falciparum* sporozoite invasion. **(A)** Inside-outside staining of *P. falciparum* sporozoites in HC-04 cells after 24 h. Red staining denotes sporozoites inside cells; yellow staining denotes sporozoites outside cells. Scale bars = 200 μm. **(B,C)** The invasion efficiency of *P. falciparum* NF54 sporozoites in HC-04 (parental) cells grown under normoxic conditions (5% CO_2_) **(B)** or in 5% oxygen **(C)**. The dashed red line at 2% indicates the highest invasion percentages reported in the literature using primary human hepatocyte monoculture. AA: amino acids (arginine, cysteine, histidine, isoleucine, leucine, lysine, methionine, phenylalanine, threonine, tryptophan, tyrosine, valine). Lipid: arachidonic, linoleic, linolenic, myristic, oleic, palmitic, stearic, cholesterol, Tween-80, tocopherol acetate, and Pluronic F-68. Glc: 15 mM D-glucose was added at the time of sporozoite addition. **(D)** The invasion efficiency of *P. falciparum* NF54 sporozoites isolated from the same pool of mosquitoes in parental HC-04 and the HC-04.J7 subclone grown in DMEM-NoGlc in three biological replicate (R1–R3) assays. Mean ± SEM is shown. *P*-values were calculated using a using a two-tailed Student’s *t*-test. ^∗∗^*P* < 0.01, ^∗∗∗^*P* < 0.001.

The original HC-04 growth media for sporozoite invasion, termed “culture media” (CM), achieved an invasion efficiency of 0.13% with *P. falciparum* sporozoites (Sattabongkot et al., 2006). When we cultured HC-04 in DMEM-NoGlc in an attempt to reduce the Warburg effect typically seen in cancer cells (Warburg et al., 1924), we observed differences in cellular morphology (Supplementary Figure S1A) and expression levels for proteins involved in oxidative phosphorylation and proteins found in the mitochondria (Supplementary Figures S1B–E and Supplementary Tables S1–S3). While HC-04 cells were invaded using both media conditions (Figure 2A), the change to DMEM-NoGlc resulted in a notable increase in the percentage of HC-04 cells invaded by *P. falciparum* sporozoites from the 0.13% originally published using CM (Sattabongkot et al., 2006) (Figure 2B,C). We also tested the addition of amino acids, the addition of a lipid mixture, Glc supplementation upon sporozoite addition (Itani et al., 2014), and the use of hypoxia (Ng et al., 2014) in the system. The addition of amino acids or Glc supplementation had little effect on the invasion rate, while the addition of the lipid mixture and the use of hypoxia produced slightly higher invasion efficiency, as well as an undesired increase in the variability of the invasion rate (Figure 2B,C).

Given the morphological differences between individual cells and the high variability in infection rates under different conditions, especially under hypoxia (Figure 2B,C), we hypothesized that HC-04 is actually a mixed population of cells. Since the HC-04 cell line used here has never been cloned (Prachumsri and Yimamnuaychok, 2002), we performed limiting dilution subcloning (Figure 1). Of the 10 clones produced, five survived (clones 2, 3, 5, 7, and 8) (Supplementary Figure S2). Initial infection studies with these five clones suggested that more sporozoites that had entered clone 7 cells (HC-04.J7) were “rounding up” or initiating LS development 24 h post-invasion. Using our optimized DMEM-NoGlc media, we compared the mean invasion rate of 1.01% (range: 0.78–1.2%) in a newly thawed stock of parental “mixed” HC-04 with the invasion rate in HC-04.J7 in an independent laboratory and observed a statistically significant improvement in the mean invasion rate to 3.3% (range: 2.5–5.1%) in the HC-04.J7 (Figure 2D).

Using an ILSDA in the HC-04.J7 line to validate this *in vitro* system, we found that higher concentrations of the 2A10 anti-CSP antibody inhibited sporozoite invasion more than lower concentrations, with 1 μg/mL of 2A10 inhibiting 77% of invasion events (Table 1). To further validate this *in vitro* system for potential use in long-term LS studies and confirm that the increase in invasion events represented *bona fide* initiation of LS development, as well as to determine whether the improved invasion percentage seen in HC-04.J7 could lead to higher levels of continued LS development vs. HC-04, we extended the assay to allow for early exoerythrocytic form development. Based on the observations that long-term (≥4 days) culture of HC-04 in DMEM-NoGlc resulted in dying cells and that the parasite requires Glc for energy production and development (Itani et al., 2014), we hypothesized that full LS maturation would not be possible in DMEM-NoGlc media and that a switch to IMDM or MEM + F12 media would be necessary to support both hepatocyte survival and LS growth and maturation. A preliminary test of this hypothesis demonstrated that sporozoite-infected HC-04 and HC-04.J7 cells can reach day 5 LS development (Supplementary Figure S3). We also noted variable LS developmental phenotypes at this time point, with both large (≈20–30 μm) and small (≈5–10 μm) exoerythrocytic forms evident in HC-04 and HC-04.J7.

**Table 1:** Inhibition of liver stage development assay.

mAb	Concentration	% Invasion of HC-04.J7 Mean (±*SEM*)	% Inhibition	*P*-value^∗^
1D9 (control)	10 μg/mL	3.70 (0.71)	–	–
2A10	3 μg/mL	1.07 (0.24)	71	<0.0001
	1 μg/mL	0.85 (0.16)	77	<0.0001
	0.3 μg/mL	1.74 (0.38)	53	0.0010
	0.1 μg/mL	1.52 (0.37)	58	0.0006

*^∗^ANOVA, Holm–Sidak’s multiple comparisons test, α = 0.05. HC-04.J7 cells were treated with the monoclonal antibody 2A10 or the isotype-matched control antibody 1D9 to block sporozoite invasion, and invasion was quantified. An ANOVA with corrections for multiple comparisons was used to compare each invasion percentage to that of the control. This is a representative experiment with at least three biological replicates per antibody concentration from two independent biological assays using two different sporozoite pools.*

Using our optimized invasion model followed by a switch to MEM + F12 media after 24 h, we observed that the percentage of HC-04 and HC-04.J7 cells containing a developing exoerythrocytic form at day 5 post-invasion (Table 2) was comparable to or even higher than the percentage containing a sporozoite at 24 h (Figure 2B–D) and 48 h post-invasion (data not shown), suggesting that the parasites can survive and progress toward schizont formation following the initial invasion and demonstrating the accuracy and reproducibility of the invasion rates observed at 24 h. Additionally, a higher percentage of HC-04.J7 cells contained a developing exoerythrocytic form compared to HC-04.

**Table 2:** Exoerythrocytic form development is supported following sporozoite invasion.

	Rep 1 MSP-1 % (N)	Rep 2 MSP-1 % (N)	Rep 3 MSP-1 % (N)	Rep 4 MSP-1 % (N)	Rep 5 MSP-1 % (N)	Rep 6 MSP-1 % (N)	HSP70 % (N)
HC-04.J7	4.2 (20)	4.2 (20)	4.9 (12)	4.7 (12)	3.7 (12)	7.4 (17)	6.9 (20)
HC-04	1.6 (20)	2.7 (20)	1.5 (12)	1.4 (12)	1.0 (12)	3.6 (17)	1.5 (19)

*%, percent infection, N = # of fields read. Rep, Biological replicate number. Merozoite surface protein-1 (MSP-1) staining with mAb 5.2-Alexa488 conjugated. P. falciparum HSP70 staining with pAb. Coverslips with HC-04.J7 or HC-04 cells were harvested and fixed 5 days post-infection and stained with anti-MSP-1 or HSP70 antibodies; the percentage of HC-04 cells containing developing exoerythrocytic forms is reported.*

### Comparative Proteomics of HepG2, HC-04, and HC-04.J7 Identified Glypican-3 as a Putative Receptor for *P. falciparum* Sporozoites

**FIGURE 3:**
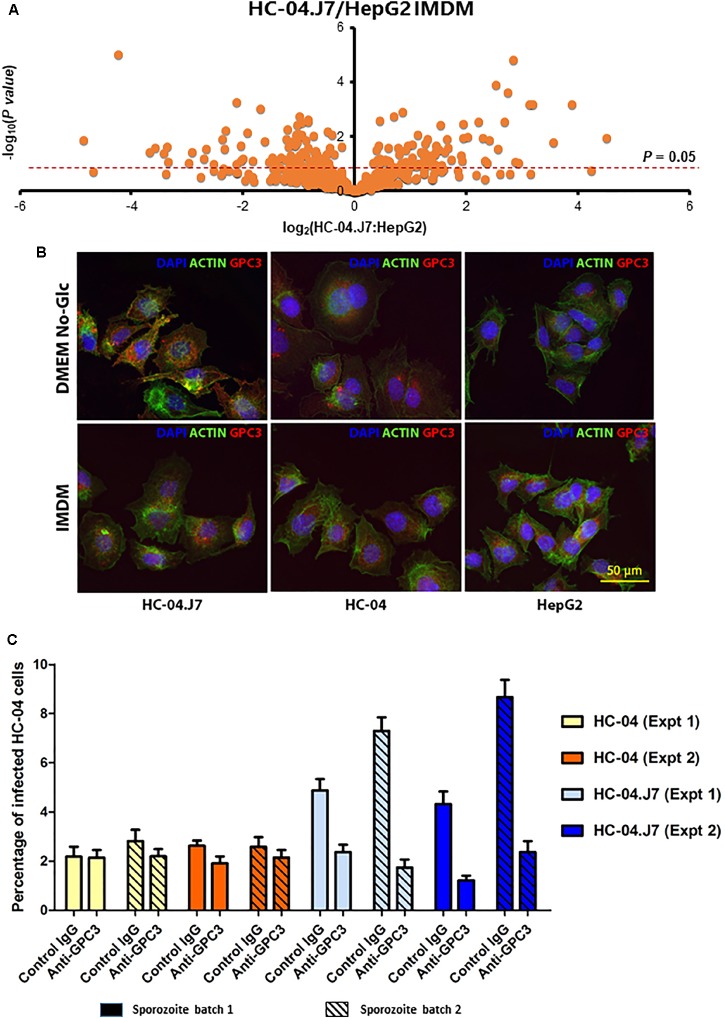
Multi-omics analysis of HC-04, HC-04.J7, and HepG2 cell lines. **(A)** Volcano plot of the quantifiable surface-enriched proteome comparing HC-04.J7 protein levels to HepG2 protein levels when both cell lines are grown in IMDM. **(B)** Immunofluorescence staining of HC-04, HC-04.J7, and HepG2 cells grown in DMEM-NoGlc or IMDM with anti-GPC3 antibody. **(C)** An inhibition of liver stage development assay with anti-GPC3 antibody. Results from two representative experiments carried out using the same methodology with two different sporozoite pools with mean ± SEM are shown. A two-way ANOVA showed a significant effect of the antibody on sporozoite invasion in HC-04.J7 (*P* < 0.001).

Considering that the parental HepG2 cell line used in this study does not support *P. falciparum* sporozoite invasion, while HC-04 and HC-04.J7 do, we hypothesized that there must be a key difference between the invasion-resistant HepG2 and the invasion-susceptible HC-04 and HC-04.J7 (Figure 1). We performed a cell membrane-targeted proteomic analysis of all three cell lines to identify potential host cell surface receptor(s) for invasion (Figure 3A). Comparative analyses of the cell lines were done using IMDM as the culture media since HepG2 growth was not supported in the DMEM-NoGlc media; in our experience, *P. falciparum* sporozoite invasion can still occur in HC-04 cells grown in IMDM (data not shown).

As expected, we found more notable differences between the HepG2 and HC-04 cell lines than between HC-04 and HC-04.J7 (Supplementary Tables S4–S6 and Supplementary Figure S4). We used *in silico* analyses to narrow the list of proteins and focused on those that (i) displayed statistically significant differences in expression between HepG2 and HC-04.J7 (due to the similarities between HC-04 and HC-04.J7 and the need to select one or the other as the initial comparator for HepG2, we chose to use the more susceptible HC-04.J7 line initially; HC-04 was used for further validation of differences between invasion-resistant and invasion-susceptible cells), (ii) had features that indicate localization to the cell membrane based on gene ontology terms, and (iii) had putative receptor functions. Using these criteria, we identified six proteins (Table 3). We selected GPC3 for further analyses as it had the largest HC-04.J7/HepG2 fold expression change of the six proteins and had been identified as upregulated in HC-04 in a previous comparison of HC-04 and HepG2 (Tao et al., 2014). GPC3 was expressed at similar levels in both HC-04 and HC-04.J7 (Figure 3B and Supplementary Table S4). Microscopic analysis suggested some residual GPC3 expression in HepG2 that was not captured by mass spectrometry (Figure 3B). We performed an ILSDA with an anti-GPC3 antibody; while anti-GPC3 had a minimal effect (19–25% reduction) on the *P. falciparum* sporozoite invasion efficiency in HC-04 cells (*P* > 0.05), it had a marked inhibitory effect (70–76% reduction) on invasion in HC-04.J7 cells (*P* < 0.001) (Figure 3C).

**Table 3:** Proteins enriched in HC-04.J7 compared to HepG2.

Identified proteins	Accession number	Molecular mass	Extracellular membrane protein?	Cell receptor?	Fold expression HC-04.J7/HepG2	*P*-value
Integrin alpha-2	ITA2_HUMAN	129 kDa	YES	YES	6.29	0.00291
**Glypican-3**	**GPC3_HUMAN**	**66 kDa**	**YES**	**YES**	**69.61**	**0.00298**
B-cell receptor-associated protein 29	BAP29_HUMAN	28 kDa	YES	YES	30.16	0.00474
Transferrin receptor protein 1	TFR1_HUMAN	85 kDa	YES	YES	3.14	0.02576
Integrin alpha-V	ITAV_HUMAN	116 kDa	YES	YES	47.89	0.02995
Transient receptor potential cation channel subfamily V member 2	TRPV2_HUMAN	86 kDa	YES	YES	18.70	0.03006

*Proteins with a fold change greater than 1.5 and a P-value < 0.05 when HC-04.J7 and HepG2 normalized spectral counts are compared that are also extracellular membrane proteins with reported receptor function. Glypican-3 was selected for further analysis and is presented in bold here. P-values were calculated using a two-tailed Student’s t-test comparing two samples with equal variance.*

### Comparative RNA-Seq of HepG2, HC-04, and HC-04.J7 Reveals Several Features Unique or Enriched in Cells That Are Susceptible to *P. falciparum* Sporozoite Invasion

**FIGURE 4:**
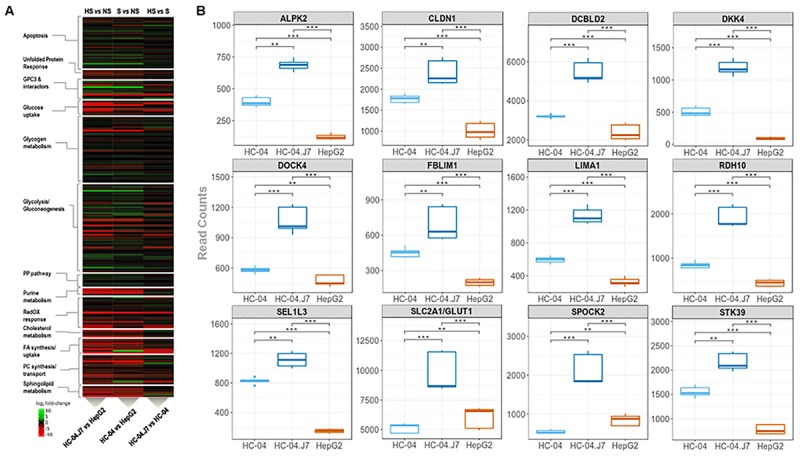
Transcriptomic mining reveals pathways and genes that partition hepatocarcinoma lines that are (i) highly susceptible, HC-04.J7, (ii) susceptible, HC-04 (parental), or (iii) non-susceptible, HepG2, to *P. falciparum* sporozoite infection. **(A)** Heatmap displaying the relative transcript reads of various transcripts comparing HC-04.J7 (highly susceptible, HS) and HepG2 (non-susceptible, NS; left); HC-04 (susceptible, S) and HepG2 (center); and HC-04.J7 and HC-04 (right). **(B)** Boxplot analyses for a subset of genes through the transcriptomic profiling of the three cell lines. Read counts are indicated along the *y*-axes for each comparison. *P*-values were calculated using a two-sided Mann–Whitney *U* test. ^∗^*P* < 0.05, ^∗∗^*P* < 0.01, ^∗∗∗^*P* < 0.001.

Our initial proteomics analysis of membrane-enriched proteins focused on the identification of novel cell surface receptors for sporozoites. However, this approach only captures the hepatocyte surface proteome at a specific time point and culture condition. To gain a more global perspective of the differences between the invasion-resistant HepG2 cell line and the invasion-susceptible HC-04 lines, we performed a global RNA-seq analysis (Figure 1 and Supplementary Table S7). As expected, in general, the differences between HC-04 and HC-04.J7 were not as notable as the differences between either HC-04 line and HepG2 (Figure 4A). *GPC3* transcript expression was significantly higher in both HC-04 and HC-04.J7 compared to HepG2, though transcript reads were found in HepG2 (Figure 4A and Supplementary Table S7). Additionally, we identified several classes of transcripts that showed significantly different levels of expression in HepG2 as compared to the HC-04s. Many of these classes were involved in cellular metabolism (Figure 4A), such as Glc uptake (*GTR9*, *PCKGC*, *G6PC*, and *GTR5*); glycogen metabolism (*PPP2R3A*, *PPP2R2C*, *GYS2*, and *PHKG1*); glycolysis (*PRKP*, *ALDOB*, *SLC2A5*, *HK1*, and *LDHAL6B*); and sphingolipid metabolism (*SPTC3* and *SPTSB*) (Supplementary Table S7). We noted upregulated expression profiles for a suite of genes related to apoptosis, the unfolded protein response, polarized cellular architecture, and hepatic regeneration in HC-04/HC-04.J7 compared to HepG2 (Figure 4A and Supplementary Table S7). Of the 105 hepatocyte genes that were observed to be differentially expressed, 28 were found to be upregulated in both HC-04 and HC-04.J7. Four of these upregulated genes, claudin (*CLDN1*), *DOCK4*, *FBLIM1*, and LIMA1 (also known as EPLIN) (*LIMA1*/*EPLIN*), are known to be important for cellular junctions (Figure 4B; Furuse et al., 1998; Brown et al., 1996; Tu et al., 2003; Abraham et al., 2015; Sundaravel et al., 2015). Additionally, two of these upregulated genes are related to the epithelial characteristics of a cell: *LIMA1*/*EPLIN* and *ALPK2* (Figure 4B; Yoshida et al., 2012; Fagerberg et al., 2014). Another two of these upregulated genes are associated with antiproliferative effects, *RDH10* and the discoidin, CUB, and LCCL domain containing protein 2 (*DCBLD2*) (Figure 4B; Rossi et al., 2007; Kim et al., 2008). Also in the set of 28 genes upregulated in HC-04.J7, there is the serine, threonine kinase *STK39*, which is involved in cellular stress responses (Johnston et al., 2000), the Glc transporter *SLCA2*/*GLUT1*, which has been previously shown to be essential for *Plasmodium* hepatic infection (Meireles et al., 2017), the proteoglycan *SPOCK2*, and *SEL1L3* (Figure 4B). Proteoglycans have been previously associated with parasite invasion (Frevert et al., 1993; Armistead et al., 2011).

### *In silico* Analyses of GPC3 Interacting Pathways Highlight Potential Players in Sporozoite Invasion

**FIGURE 5:**
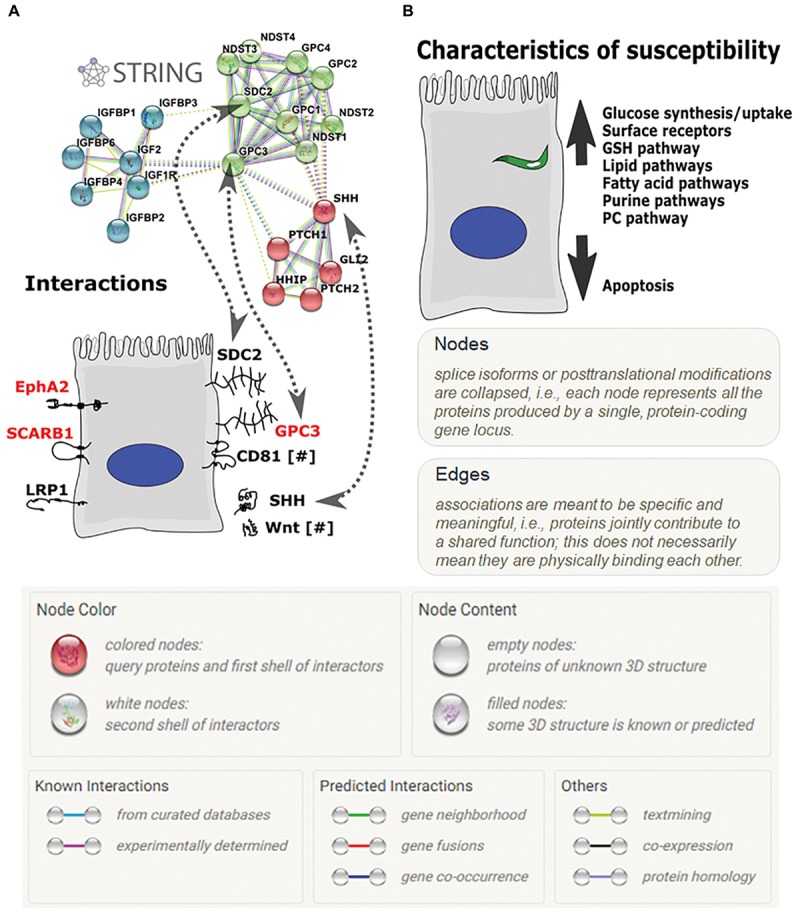
Hepatocyte receptors and pathways involved in *P. falciparum* sporozoite invasion. **(A)** The glypican-3 (GPC3) STRING protein–protein interaction network. Proteins on the right side of the hepatocyte include GPC3 and its known interactors; proteins on the left side are other hepatocyte receptors implicated in sporozoite invasion. [GPC, glypican; NDST, *N*-deacetylase/*N*-sulfotransferase (heparan glucosaminyl); IGFBP, Insulin-like growth factor binding protein 4; IGF, insulin-like growth factor; IGF1R, Insulin-like growth factor 1 receptor; PTCH, patched; HHP, Hedgehog interacting protein; GLI2, GLI family zinc finger 2; purple lines—experimentally determined interactors; light blue lines—curated database-determined interactors; light green lines—text mining-determined interactors; black lines—co-expression-determined interactors]. **(B)** Characteristics of a more-susceptible hepatocyte for *P. falciparum* invasion based on proteomic and transcriptomic analyses.

Further analyses of GPC3 and its interacting pathways using STRING protein–protein interactions suggest that other major pathways, such as Wnt and Shh signaling, may play key roles in parasite invasion (Figure 5A). Wnt and Shh both play roles in cell survival and proliferation (Logan and Nusse, 2004; Simpson et al., 2009). GPC3 and DKK4 act as Wnt pathway inhibitors (Bazzi et al., 2007; Capurro et al., 2014) and are more highly expressed in HC-04.J7 than in HepG2 (Table 3 and Figure 4A,B), suggesting that Wnt signaling inhibition may influence sporozoite host cell susceptibility.

Additionally, GPC3’s interaction with the previously identified sporozoite receptor CD81 (Silvie et al., 2003; Liu et al., 2009) downregulates cell proliferation through interaction with the hedgehog pathway (Bhave et al., 2013). This, combined with our RNA-seq data showing the upregulation of genes in the invasion-susceptible cells that play a role in inhibiting cell proliferation, *RDH10* and *DCBLD2* (Figure 4B), and the inverse correlation between HC-04 cell density and sporozoite invasion (Supplementary Figure S5), highlights the role of cell proliferation downregulation for optimal invasion.

### Previously Identified Hepatocyte Receptors for Sporozoite Invasion Show Varied Expression in HC-04.J7

Contrary to what was expected, many of the previously identified cell surface receptors involved in *P. falciparum* sporozoite invasion of hepatocytes were not identified in our HC-04.J7 cell line (Table 4). While SCARB1 (Rodrigues et al., 2008), SDC2 (Frevert et al., 1993), and EphA2 (Kaushansky et al., 2015) displayed significant transcript reads, their protein expression was very low or even undetectable in our membrane-enriched proteomic analysis. However, by microscopy, notable EphA2 protein expression was observed (Supplementary Figure S6). LRP1 (Shakibaei and Frevert, 1996) and CD81 (Silvie et al., 2003) displayed low transcript read numbers and very low or undetectable protein expression (Table 4).

**Table 4:** Previously identified hepatocyte surface receptors involved in sporozoite invasion and their expression in HC-04.J7.

Receptor	Previous citation	HC-04.J7 expression
LRP1	Shakibaei and Frevert, 1996	Proteome—0 Transcriptome—0.44
CD81	Silvie et al., 2003	Proteome—ND Transcriptome—0.11
SDC2	Frevert et al., 1993	Proteome—ND Transcriptome—2387.78
SCARB1	Rodrigues et al., 2008	Proteome—0.85 Transcriptome—1803.56
EphA2	Kaushansky et al., 2015	Proteome—0.12 Transcriptome—721.56
GPC3	Not applicable	Proteome—6.98 Transcriptome—14577.56

*Red denotes proteins with detectable expression in the HC-04.J7 membrane-enriched proteome. (LRP1: low density lipoprotein receptor-related protein, SDC2: syndecan-2, SCARB1: scavenger receptor class B member 1, EphA2: EPH receptor A2, GPC3: glypican-3)*.

## Discussion

Although questions remain regarding the complete repertoire of molecular mechanisms involved in *P. falciparum* LS biology, we have identified GPC3 as a hepatocyte receptor putatively involved in sporozoite invasion. Interestingly, a previous study showed the GPC3 heparan sulfate proteoglycan was upregulated at the transcript level in HepG2-A16 cells upon infection with irradiated sporozoites. HepG2-A16 cells represent a distinct HepG2 sub-line that differs from the parental line used in this analysis in that it is susceptible to *P. falciparum* sporozoite invasion but still does not support further parasite development (Chattopadhyay et al., 2011). This further supports the potential role of GPC3 in invasion. Based on the extensive interactions of GPC3 with the Wnt and Shh signaling pathways (Figure 5A), among others, altering GPC3 expression through over-expression or knockout constructs may have significant global effects on the overall cellular response with unforeseen impacts on sporozoite invasion, making the ILSDA with anti-GPC3 antibodies the most straightforward approach to evaluate its specific role in invasion at this time. Furthermore, while we did not find GPC3 in HepG2 cells through our membrane-enriched proteomics, we did note *GPC3* transcript reads in HepG2 and protein expression by microscopy (Figure 3B), suggesting that the protein may be expressed at levels undetectable by LC-MS, expressed in the soluble fraction rather than the membrane fraction, or translationally repressed in our culture conditions. Additional studies ectopically expressing GPC3 on the surface in HepG2 will be important for further validating the role of GPC3 as a sporozoite receptor for invasion.

The incomplete inhibition of *P. falciparum* sporozoite invasion by anti-GPC3 antibodies in HC-04 suggests that GPC3 is not the only receptor present to facilitate sporozoite invasion. This is not surprising, as redundancy among receptors has been previously shown with CD81 and SCARB1 for *P. berghei* invasion (Manzoni et al., 2017). EphA2, which has been previously shown to act as a receptor for sporozoite invasion (Kaushansky et al., 2015), is also expressed in HC-04 and HC-04.J7 (Supplementary Figure S6), and may play a role in facilitating invasion in the presence of anti-GPC3 antibodies. Future studies inhibiting both GPC3 and EphA2 will be needed to determine whether these receptors act with functional redundancy.

While the role of GPC3 as a cancer marker and during liver proliferation, regeneration, and repair has been described (Jakubovic and Jothy, 2007; Liu et al., 2009; Liu et al., 2010), GPC3 is not canonically thought to be expressed in adult hepatocytes at homeostasis (Capurro et al., 2003; Yamauchi et al., 2005). However, one study noted low levels of GPC3 protein expression by immunohistochemistry in normal human liver sections (Sung et al., 2003), and a growing body of literature supports the hypothesis that at homeostasis, the liver undergoes constant renewal and repair with hepatocytes acting as the proliferating source of new hepatocytes (Malato et al., 2001; Zhang et al., 2003). These findings suggest that GPC3 is expressed and may have previously unrecognized roles in the liver during homeostasis.

The inherent bias of LC-MS-based methods in detecting only a subset of the total proteome as a result of sample preparation method, ion suppression, and inability to amplify proteins prior to detection makes it possible to miss proteins that are present (often at low abundance) that potentially play a role in parasite invasion. This bias may have had an impact on our ability to detect the previously identified hepatocyte receptors for sporozoite invasion. Therefore, we performed an RNA-seq analysis in concert with the proteomic analyses to add a more global perspective of the invasion-resistant HepG2 cell line and the invasion-susceptible HC-04 lines (Supplementary Table S7), highlighting the potential importance of cellular junctions, epithelial characteristics, and Glc transport capabilities for successful invasion (Figure 4). It is important to note that some of these characteristics may actually play a role in parasite development rather than invasion, but this cannot be assessed in HepG2. The RNA-seq datasets generated in this study serve as a starting point for the development of future hypotheses related to sporozoite host cell selection and potential invasion blocking mechanisms, paving the way for the screening of inhibitors of *in vivo* LS development and gene knock-down experiments to assess the role of the specific candidates in invasion and/or LS development. Based on our data, we suggest that the ideal host cell is one that upregulates its metabolic capabilities while downregulating its apoptotic tendencies (Figure 5B). While the details of these mechanisms remain to be determined, interference with these host cell characteristics could provide a means of preventing *P. falciparum* infection.

Previous *Plasmodium* LS studies have suffered from significant technical limitations. *In vivo, P. falciparum* can infect mice with chimeric human livers, but use of these mice is fiscally prohibitive for many laboratories (Sacci et al., 2006; Vaughan et al., 2012). *In vitro, P. falciparum* infects primary human hepatocytes, but with highly variable infection rates (0.13–2%) (Smith et al., 1984; Mazier et al., 1985; Vaughan et al., 2008; Roth et al., 2018). Other efforts to improve *P. falciparum* LS culture have relied heavily on primary hepatocytes rather than a cell line and often involved bioengineered co-culture platforms, again limiting implementation of these systems to select laboratories (Millet and Collins, 1989; Kebaier and Vanderberg, 2010; March et al., 2013; Ng et al., 2014). While a recent LS model was developed using cryopreserved primary hepatocytes in a simple monoculture system, the requirement for hepatocyte lot screening to identify those that support sporozoite invasion and development still limits its widespread implementation (Roth et al., 2018). Our invasion model was developed to overcome the challenges of primary cells by using a homogenous, immortalized cell line that does not require lot screening and mimicking *in vivo* environmental parameters to achieve *P. falciparum* invasion rates equal to and greater than those achieved in primary hepatocyte systems (March et al., 2013; Ng et al., 2014; Roth et al., 2018). Our system represents a 20-fold increase in the invasion rate of the immortalized HC-04 cell line from that originally published (Sattabongkot et al., 2006) and a 3- to 5-fold increase from current hepatocarcinoma invasion models (Mikolajczak et al., 2011; Tao et al., 2014). Invasion was inhibited by anti-CSP antibodies, with higher concentrations of antibody inhibiting invasion more than lower concentrations (Table 1), suggesting that the invasions are natural and not artificially induced by any component of the culture system. Furthermore, by subcloning HC-04.J7, we identified an HC-04 subclone with an increased susceptibility to sporozoite invasion (Figure 2D) and the ability to support early exoerythrocytic form development (Supplementary Figure S3 and Table 2).

Beyond the implications for the study of *P. falciparum* liver invasion, this study demonstrates the utility of mimicking *in vivo* conditions to restore more organ-like features of cell lines. Many immortalized cell lines undergo the Warburg effect in metabolism and adopt properties very different from those of their parent tissue (Warburg et al., 1924). By mimicking *in vivo* conditions, these cells can recover some of their more natural properties, evident in a shift toward oxidative phosphorylation and the citrate cycle in HC-04 cells (Supplementary Figures S1C,D and Supplementary Table S1).

Overall, this infection platform for the study of the *P. falciparum* LS and inhibition thereof could be applied to the study of other *Plasmodium* species, like *P. vivax*. Moreover, this system can potentially supplant current ILSDA approaches as a cost-effective platform for screening antibodies or small molecule inhibitors that prevent sporozoite invasion and LS development. A limitation of the current culture system is the description of LS phenotypes up to day 5 and the lack of a method to permit the infection of red blood cells by mature merozoites. However, the data sets described serve as both a valuable resource and launching point for the greater scientific community to spur the development and testing of new hypotheses in the context of *Plasmodium* LS biology as well as to explore further development of an *in vitro* method to bridge the LS and asexual development in red blood cells, ultimately achieving the completion of the human life cycle of *P. falciparum in vitro*.

## Author Contributions

RT, JK, EB-L, and RD conceptualized the study. RT, JK, DT, TH, and RD developed the methodology. RT, JK, and TR carried out the invasion assays. RT, TR, and EB-L performed the invasion quantifications. TR and EB-L performed the ILSDAs. RT and AN performed the microscopy. DT, TH, RT, and HL performed the proteomic analyses and data curation. RT and TH performed the RNA extraction. SL, AG, JB, and JK performed the RNA-seq read assembly and bioinformatics data analysis. RD provided oversight of the study. RT and RD drafted the manuscript. JK was involved in data visualization and preparation of figures. All authors contributed to the final writing’s review and editing.

## Conflict of Interest Statement

The authors declare that the research was conducted in the absence of any commercial or financial relationships that could be construed as a potential conflict of interest.

## Footnotes

1 https://string-db.org/

## Abbreviations

ACN: acetonitrile
ALPK2: alpha kinase 2
BSA: bovine serum albumin
CLDN1: claudin
CM: culture media
CSP: circumsporozoite protein
DCBLD2: discoidin, CUB, and LCCL domain containing protein 2
DKK4: dickkopf 4
DMEM: Dulbecco’s Modified Eagle Medium
DMEM-NoGlc: glucose-free Dulbecco’s Modified Eagle Medium
DOCK4: dedicator of cytokinesis
EphA2: EPH receptor A2
EPLIN: epithelial protein lost in neoplasm
FA: formic acid
FASP: filter-aided sample preparation
FBLIM1: filamin-binding LIM protein 1
Glc: glucose
GPC3: glypican-3
HIFBS: heat-inactivated fetal bovine serum
ILSDA: inhibition of liver stage development assay
LIMA1: LIM domain and actin-binding protein
LRP1: low density lipoprotein receptor-related protein
LS: liver stage
MEM: minimum essential medium
RDH10: retinol dehydrogenase 10
SCARB1: scavenger receptor class B member 1
SDC2: syndecan-2
SEL1L3: sel-1 suppressor of lin-12-like family member 3
Shh: Sonic Hedgehog
SPOCK2: sparc/osteonectin, cwcv, and kazal-like domain
