# Supplementary material for: The Selection of a Hepatocyte Cell Line Susceptible to Plasmodium falciparum Sporozoite Invasion That Is Associated With Expression of Glypican-3
Source: Front Microbiol. 2019 Feb 28;10:127. doi: 10.3389/fmicb.2019.00127 (PMC6413710; doi:10.3389/fmicb.2019.00127)
Supplement: Supplementary file 8 [file Data_Sheet_1.PDF]

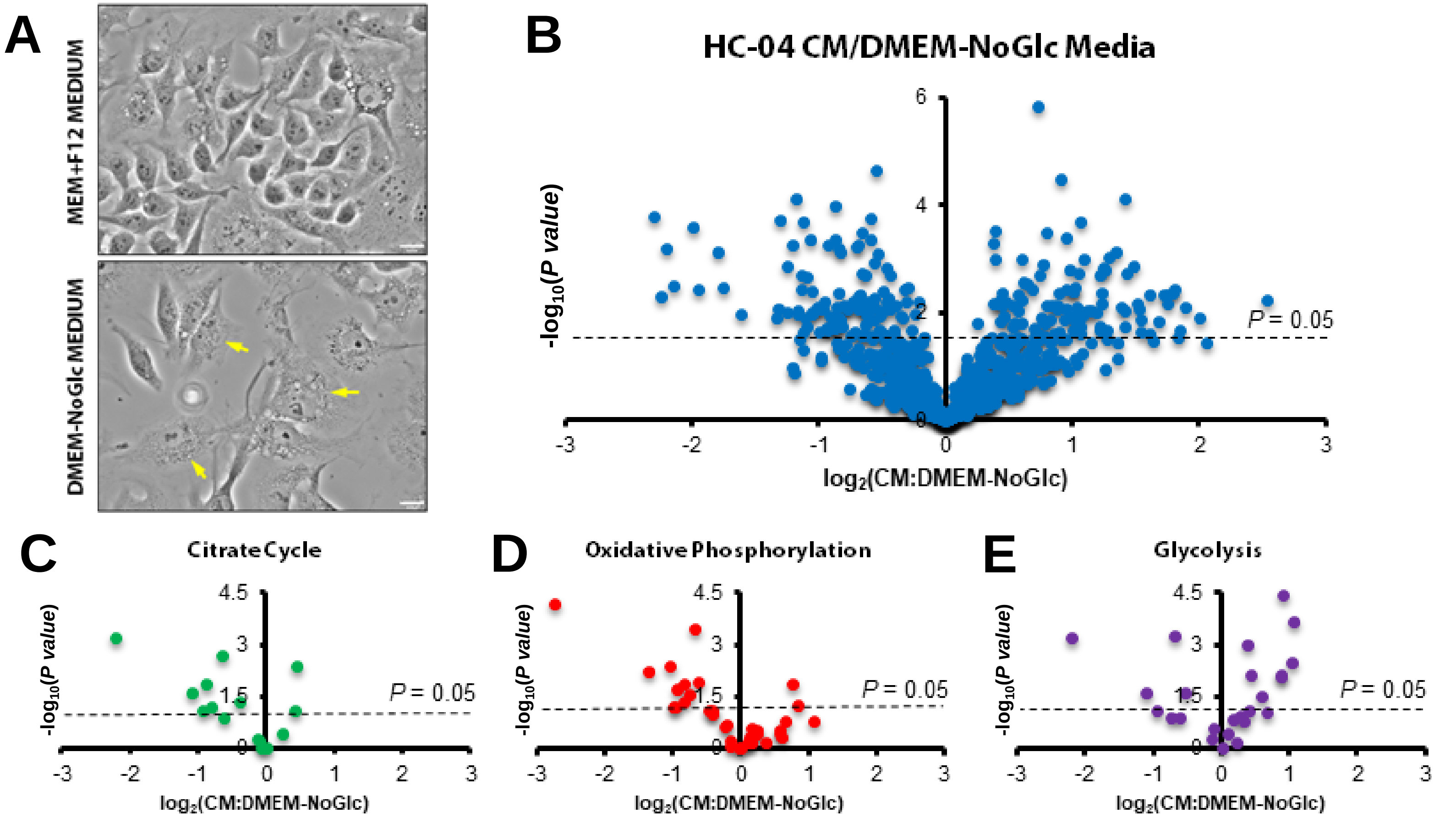

Supplementary Figure 1. Global proteomic analysis of HC-04 cells in different culture media informs the development of an optimized protocol for enhanced invasion efficiency of *Plasmodium falciparum*. (A) Morphology of HC-04 cells grown in the original MEM+F12 culture media (CM) vs DMEM-NoGlc. Scale bars = 16 µm. **(B)** Volcano plot of quantifiable proteome of HC-04 protein levels when grown in CM vs DMEM-NoGlc. **(C-E)** Volcano plots of the subset of proteins that fall within the given pathways in HC-04 grown in CM vs DMEM-NoGlc.

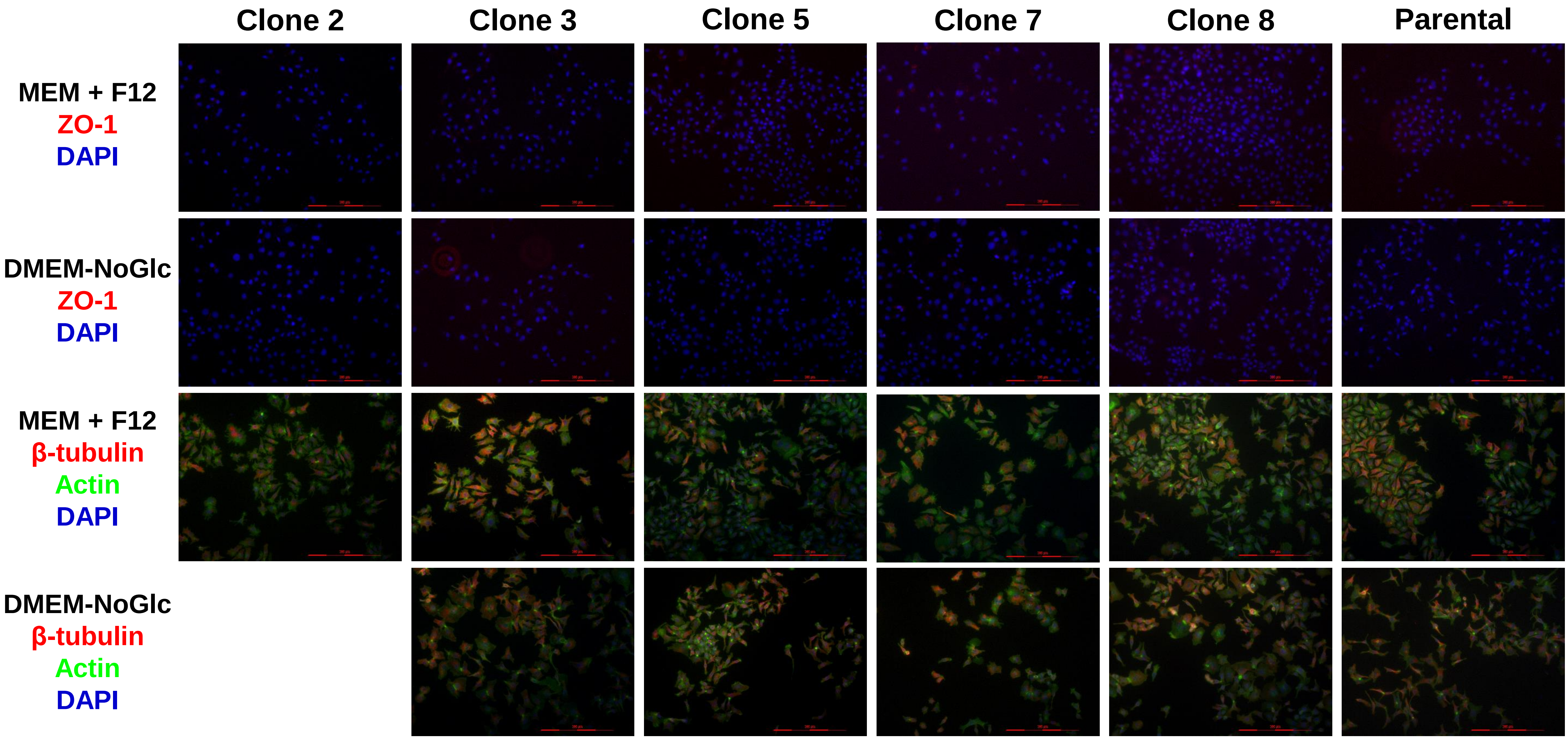

Supplementary Figure 2. Tight junction and cytoskeletal staining of HC-04 clones from limited dilution sub-cloning grown in MEM + F12 culture medium and DMEM-NoGlc medium. HC-04 cells (parental or subclones 2, 3, 5, 7, and 8) were grown for 24 hours in DMEM-NoGlc or culture medium and stained with ZO-1 and DAPI to show tight junction formation or stained with β-tubulin, actin, and DAPI to show the cytoskeleton. Sub-clones showed slightly different staining and morphology from the parental line and from other sub-clones. Scale bar = 300 µm. Clone 2 did not survive well in DMEM-NoGlc medium, so cytoskeletal staining was not performed.

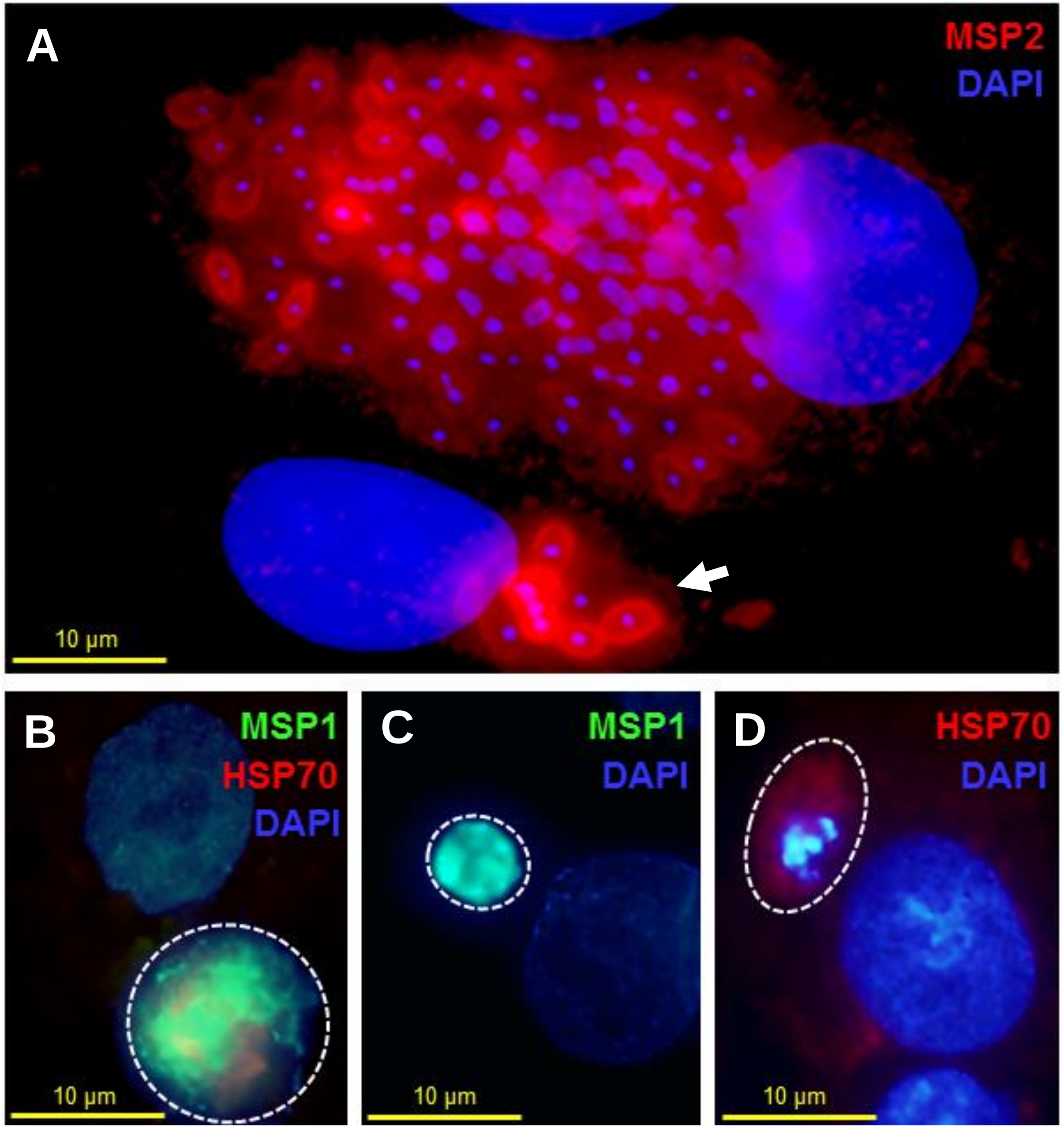

Supplementary Figure 3. *Plasmodium falciparum* exoerythrocytic form development in HC-04 and HC-04.J7. (A) *P. falciparum* exoerythrocytic forms, day 5 post-invasion in HC-04 cells grown in MEM + F12. Representative extended depth of field image of MSP2 staining of individual exoerythrocytic forms within two hepatocytes are shown (a large one on top and a smaller one below, arrow). The image was acquired using confocal microscopy, and image stacks were deconvoluted prior to combining and focused along the plane of the parasites. **(B-D)** *P. falciparum* exoerythrocytic forms, day 5 post-invasion in HC-04.J7 grown in DMEM-NoGlc and switched to MEM + F12 during development. Note that while sporozoite invasion has been optimized, full exoerythrocytic form development has not, giving rise to variable developmental phenotypes at this time point that can be readily observed and scored in the culture system. Each was considered as a positive when scoring for infection at day 5 post-sporozoite invasion. Images were acquired using epifluorescence microscopy to enable high-throughput scoring and image acquisition. Representative HSP70 and MSP1 images at a single focal plane for replicate cultures are shown. HSP70 and MSP1 were used to score either in tandem **(B)** or independently **(C,D)**, as HSP70 and MSP1 can capture earlier and later developmental transitions, respectively. Dotted lines demarcate exoerythrocytic form boundaries. Scale bar = 10 µm.

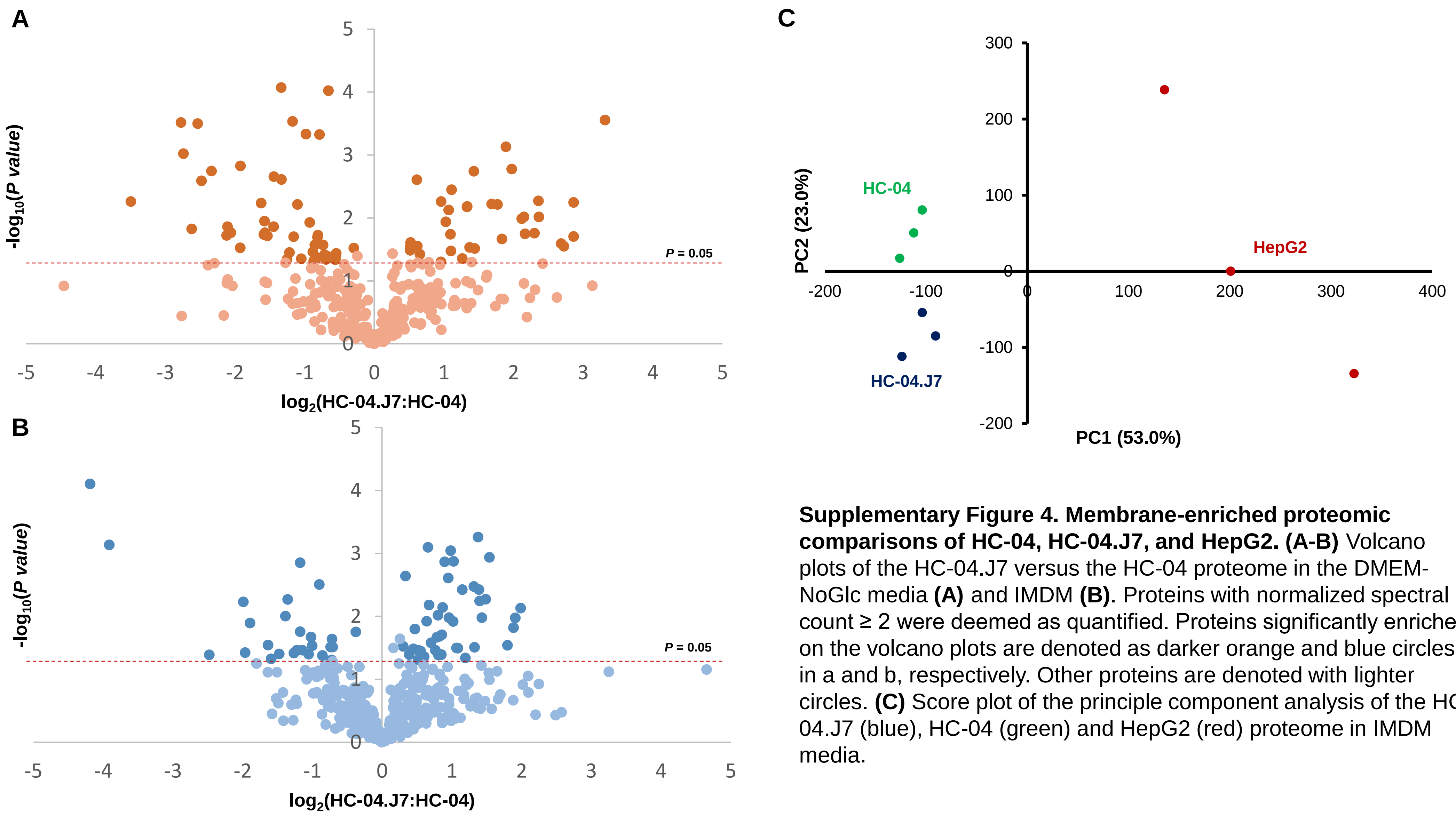

Supplementary Figure 4. Membrane-enriched proteomic comparisons of HC-04, HC-04.J7, and HepG2. (A-B) Volcano plots of the HC-04.J7 versus the HC-04 proteome in the DMEM-NoGlc media **(A)** and IMDM **(B)**. Proteins with normalized spectral count ≥ 2 were deemed as quantified. Proteins significantly enriched on the volcano plots are denoted as darker orange and blue circles in a and b, respectively. Other proteins are denoted with lighter circles. **(C)** Score plot of the principle component analysis of the HC-04.J7 (blue), HC-04 (green) and HepG2 (red) proteome in IMDM media.

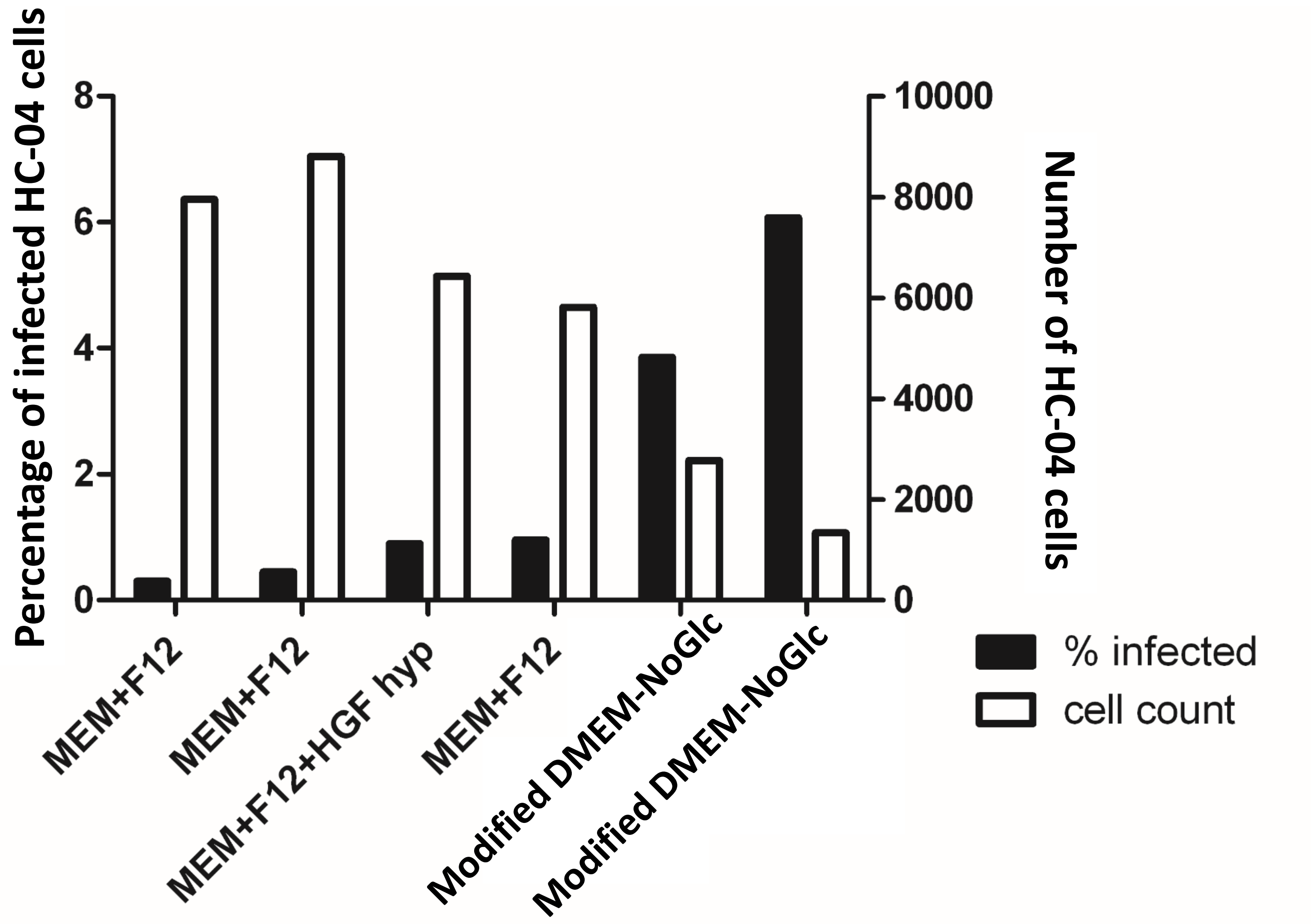

Supplementary Figure 5. Correlation between HC-04 cell density and sporozoite invasion percentage. HC-04 were grown for 24 hours in MEM+F12 CM (with or without hepatocyte growth factor (HGF) under normoxic or hypoxic (hyp) conditions) or modified DMEM-NoGlc media (supplemented with 10% HIFBS, amino acids, and lipids), then infected with *P. falciparum* sporozoites for 24 hours. The total number of cells and the number of cells infected with a sporozoite in the counted zone were recorded.

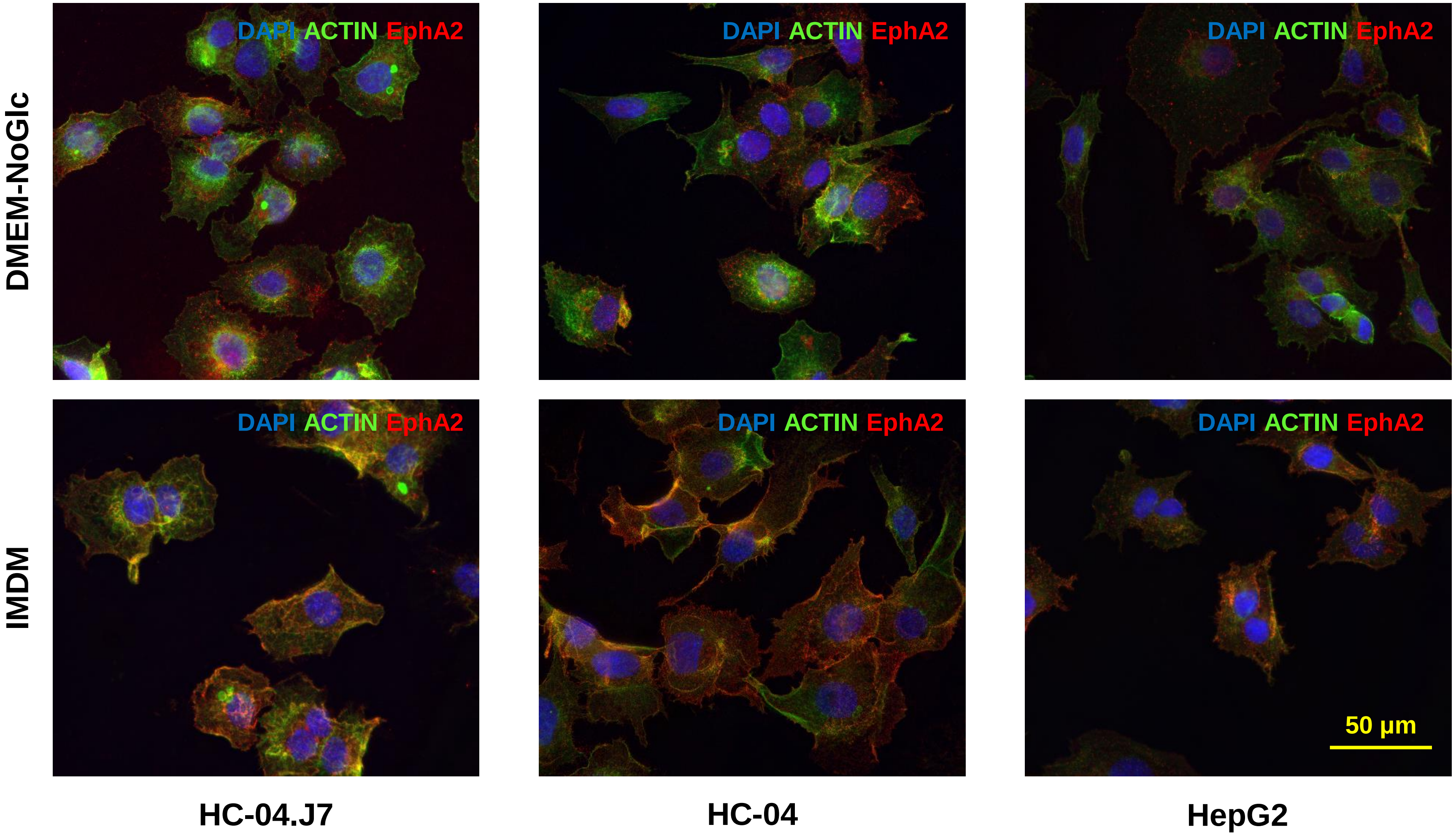

Supplementary Figure 6. Immunofluorescence staining of HC-04, HC-04.J7, and HepG2 cells with anti-EphA2 antibody in DMEM-NoGlc or IMDM. Scale bars represent 50 µm.
